# Low Mannitol Concentrations in *Arabidopsis thaliana* Expressing *Ectocarpus* Genes Improve Salt Tolerance

**DOI:** 10.3390/plants9111508

**Published:** 2020-11-07

**Authors:** Pramod Rathor, Tudor Borza, Yanhui Liu, Yuan Qin, Sophia Stone, Junzeng Zhang, Joseph P. M. Hui, Fabrice Berrue, Agnès Groisillier, Thierry Tonon, Svetlana Yurgel, Philippe Potin, Balakrishnan Prithiviraj

**Affiliations:** 1 Marine Bioproducts Research Laboratory, Department of Plant, Food and Environmental Sciences, Dalhousie University, Truro, NS B2N 5E3, Canada; pramod.rathor@dal.ca (P.R.); tudor.borza@dal.ca (T.B.); syurgel@dal.ca (S.Y.); 2 Fujian Provincial Key Laboratory of Haixia Applied Plant Systems Biology, State Key Laboratory of Ecological Pest Control for Fujian and Taiwan Crops, Center for Genomics and Biotechnology, College of Life Science, Fujian Agriculture and Forestry University, Fuzhou 350002, China; 2180514010@fafu.edu.cn (Y.L.); yuanqin@fafu.edu.cn (Y.Q.); 3 Department of Biology, Dalhousie University, Halifax, NS B3H 4R2, Canada; s.stone@dal.ca; 4 Aquatic and Crop Resource Development Research Centre, National Research Council of Canada, Halifax, NS B3H 3Z1, Canada; junzeng.zhang@nrc-cnrc.gc.ca (J.Z.); Joseph.Hui@nrc-cnrc.gc.ca (J.P.M.H.); Fabrice.Berrue@nrc-cnrc.gc.ca (F.B.); 5 Unité Fonctionnalité et Ingénierie des Protéines (UFIP), UMR 6286 CNRS, Université de Nantes, 44322 Nantes, France; agnes.groisillier@univ-nantes.fr; 6 Centre for Novel Agricultural Products, Department of Biology, University of York, Heslington YO105DD, UK; thierry.tonon@york.ac.uk; 7 Sorbonne Université, CNRS, UMR 8227, Integrative Biology of Marine Models (LBI2M), Station Biologique, 29680 Roscoff, France; philippe.potin@sb-roscoff.fr

**Keywords:** mannitol biosynthesis genes, mannitol-1-phosphate dehydrogenase, mannitol-1-phosphatase, *Ectocarpus* sp., *Arabidopsis thaliana*, abiotic stress tolerance, salt stress

## Abstract

Mannitol is abundant in a wide range of organisms, playing important roles in biotic and abiotic stress responses. Nonetheless, mannitol is not produced by a vast majority of plants, including many important crop plants. Mannitol-producing transgenic plants displayed improved tolerance to salt stresses though mannitol production was rather low, in the µM range, compared to mM range found in plants that innately produce mannitol. Little is known about the molecular mechanisms underlying salt tolerance triggered by low concentrations of mannitol. Reported here is the production of mannitol in *Arabidopsis thaliana*, by expressing two mannitol biosynthesis genes from the brown alga *Ectocarpus* sp. strain Ec32. To date, no brown algal genes have been successfully expressed in land plants. Expression of mannitol-1-phosphate dehydrogenase and mannitol-1-phosphatase genes was associated with the production of 42.3–52.7 nmol g^−1^ fresh weight of mannitol, which was sufficient to impart salinity and temperature stress tolerance. Transcriptomics revealed significant differences in the expression of numerous genes, in standard and salinity stress conditions, including genes involved in K^+^ homeostasis, ROS signaling, plant development, photosynthesis, ABA signaling and secondary metabolism. These results suggest that the improved tolerance to salinity stress observed in transgenic plants producing mannitol in µM range is achieved by the activation of a significant number of genes, many of which are involved in priming and modulating the expression of genes involved in a variety of functions including hormone signaling, osmotic and oxidative stress, and ion homeostasis.

## 1. Introduction

Plants are sessile organisms constantly subjected to abiotic stressors such as soil salinity and temperature extremes, which are considered the major limiting factors to agricultural productivity, posing a risk to global food security. Improving plant tolerance to these abiotic factors is critical for agricultural output and environmental sustainability. In the last decades, several varieties with improved tolerance to salt stress have been developed; however, the progress has been generally slow because of the multigenic nature of the traits involved in abiotic stress tolerance [1].

To achieve tolerance to salinity and drought plants produce different osmolytes including sugar alcohols, proline, polyamines, glycine betaine and proteins from the late embryogenesis abundant (LEA) superfamily [2]. Mannitol is an ubiquitous sugar alcohol found in a wide range of living organisms including bacteria, fungi, algae, and in over 100 plant species, where it serves as a metabolite, osmolyte and anti-oxidant [3,4]. Many important crop plants, however, do not produce mannitol [5]. In the last 25 years a significant number of mannitol-producing transgenic plants have been generated, using genes of very different origin: *mannitol-1-phosphate dehydrogenase* (*M1PDH*) from *Escherichia coli* and *mannose-6-phosphate reductase* (*M6PR*) from celery (*Apium graveolens*) [5,6,7,8,9,10,11,12,13,14]. These two different choices for the origin of the genes used to generate transgenic plants reflect the fact that mannitol biosynthesis and catabolism is different in the abovementioned groups of organisms. In *E. coli*, mannitol is interconverted in two steps—phosphorylation and oxidation—to fructose 6-phosphate (F6P), the later step involving M1PDH, the product of *mtlD* gene. In land plants, the interconversion of F6P to mannitol involves mannose as an intermediate in both, the mannitol biosynthetic pathway and the catabolic pathway. In the former pathway, mannose-6-P and mannitol-1-P are produced in reactions catalyzed by phosphomannose isomerase (PMI) and M6PR, respectively. In the last step, mannitol-1-phosphate phosphatase (M1Pase) will generate mannitol. The catabolic pathway proceeds through the formation of mannose and mannose-6-P, in reactions catalyzed by mannitol dehydrogenase, a hexokinase (HX) and PMI. *E.coli mtlD* was introduced into a multitude of plants, in which mannitol is not naturally produced, including important crops such as rice, maize, tomato, eggplant, peanut and tobacco, as well as the model plant *Arabidopsis thaliana*. The transgenic plants accumulated mannitol and most of them exhibited improved tolerance to salinity stress, in spite of the fact that mannitol concentration in these plants was much lower (generally between 0.1–10 µmol g^−1^ fresh weight) than in plants that naturally produce this polyol (>100 µmol g^−1^ fresh weight) [3,5,6,11,12,13,14]. Celery *M6PR* gene was introduced into *A. thaliana* and mannitol concentrations in the range of 0.5–6 µmol g^−1^ fresh weight resulted in enhanced salt tolerance [13]. Transcriptomics (microarray) analysis of mannitol-producing transgenic *A. thaliana*, carried out under standard and salinity stress conditions, revealed a wide array of pathways being differentially expressed, suggesting that mannitol-enhanced stress tolerance is achieved by global changes in gene expression and not only by increased expression of stress-inducible genes [6]. Noteworthy, none of the mannitol-producing transgenic plants, generated using *E.coli mtlD* or celery *M6PR*, expressed the phosphatase required to generate mannitol from mannitol-1-phosphate; it has been suggested that a non-specific endogenous phosphatase can perform this essential dephosphorylation step [11].

During the evolution of Eukarya, the stramenopiles, the lineage that encompasses brown algae (*Phaeophyceae*) [15], have been evolving for over a billion years independently compared with the most commonly studied multicellular eukaryotes comprising non-photosynthetic opisthokonts (including animals and fungi) and archaeplastida (including red algae and green plants) [4]. Large biomass of brown algae is found in the intertidal zone, which is a very harsh environment, with constant fluctuating levels of salinity and temperature. Mannitol is the main photosynthetate in brown algae, which includes seaweeds such as kelp (*Macrocystis* spp. and *Laminaria* spp.), rockweed (*Ascophyllum nodosum*), and *Sargassum.* In these algae, mannitol is the main carbon storage molecule as well as an osmolyte with anti-oxidant potential [4] In late summer, in some kelps, mannitol accounts for up to 25% of dry biomass [16]. To enable functional studies of brown algae, strain Ec32 of the small filamentous alga *Ectocarpus* sp., formerly included in *Ectocarpus siliculosus* [17], has been established as a genetic and genomic model [15]. In the filamentous alga *Ectocarpus sp*. mannitol content reaches 3.6–6.4% of the dry weight [18]. In brown algae, mannitol metabolism involves two enzymes for biosynthesis and two for recycling. Enzymes for biosynthesis includes M1PDH and M1Pase while mannitol recycling includes mannitol-2-dehydrogenase and a HX. The genome of *Ectocarpus* sp. Ec32 encodes three putative *M1PDH* genes (*EsM1PHD1*, *EsM1PDH2* and *EsM1PDH3*) and two *M1Pases* (*EsM1Pase1* and *EsM1Pase2*). *EsM1PHD1* (EC 1.1.1.17) and *EsM1Pase2* (EC 3.1.3.22) were overproduced in *E.coli* and characterized biochemically [19,20].

To date, no brown algal genes have been successfully expressed in land plants. Here we report the expression of *EsM1PHD1* and *EsM1Pase2* genes from *Ectocarpus* sp. Ec32 in *A. thaliana*. Transgenic plants showed improved tolerance to salinity and temperature stress, though mannitol concentrations were lower than those reported in transgenic plants expressing *E.coli mtlD* or celery *M6PR* genes. Transcriptomics analysis comparing mannitol-producing plants to wild type plants, grown under standard and salt stress conditions, revealed a significant number of differentially expressed genes involved in hormonal regulation and ABA signaling, defense mechanisms and ROS signaling, plant development, photosynthesis, K^+^ homeostasis and secondary metabolism. These findings represent a major contribution to the deciphering of the molecular mechanisms through which low concentrations of mannitol improve plant salt tolerance.

## 2. Results

### 2.1. M1PDH1 and M1Pase2 Expression and Mannitol Content in A. thaliana Transgenic Lines

The expression of *mannitol-1-phosphate dehydrogenase (M1PDH1)* and *mannitol-1-phosphatase* (*M1Pase2)* in each of the three selected independent double transgenic lines, henceforth named *Es*M1, *Es*M2 and *Es*M3, in which selection was done until the 5th generation, was determined by real-time quantitative polymerase chain reaction (qPCR) (Supplementary Table S1). Relative gene expression among the transgenic lines varied 3-fold in the case of *M1PDH1* and 2-fold in the case of *M1Pase2*. Transgenic line *Es*M1 showed the overall highest expression while *Es*M3 the lowest (Supplementary Figure S1). Gene expression was relatively high for both *M1PDH1* and *M1Pase2*, the former having a Ct value at 2–4 cycles difference from actin, the reference gene, while the later at 3–4 cycles. The content of mannitol in the transgenic lines, determined by LC-MS, was found to vary between 42.3 and 52.7 nmol g^−1^ fresh weight (Supplementary Table S2).

### 2.2. Mannitol Producing Lines Showed Enhanced Tolerance to Salinity and High Temperature Stress

**Figure 1:**
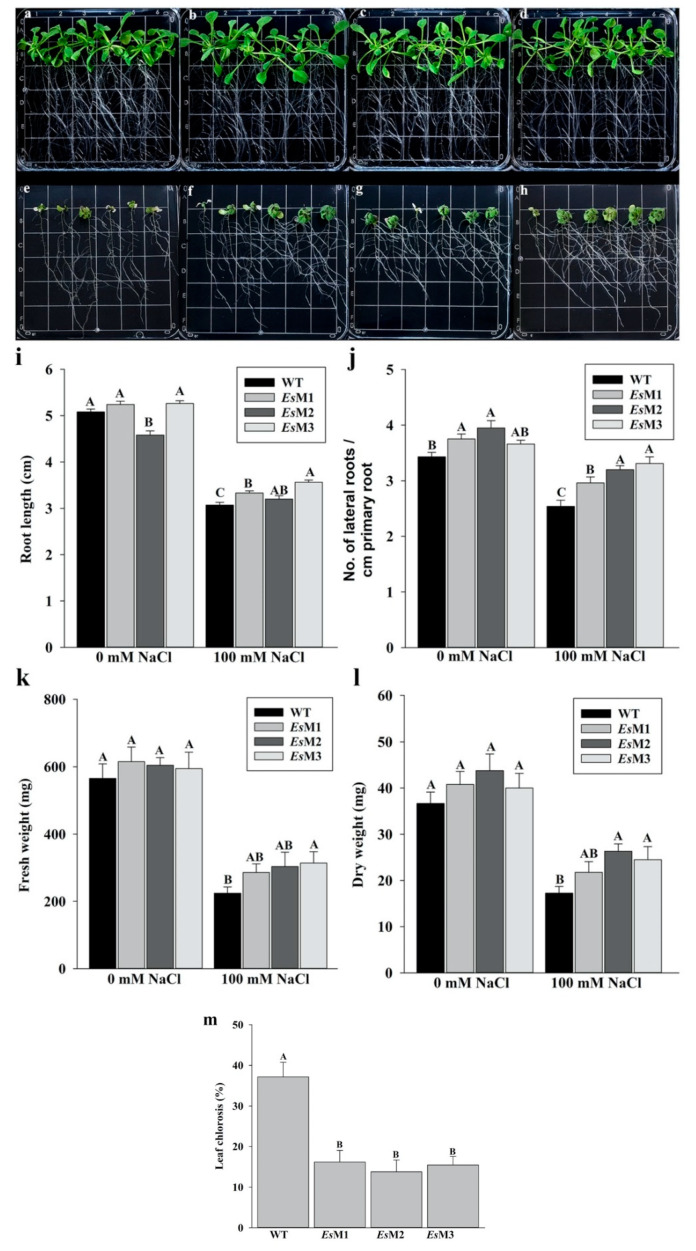
Seedlings growth, root length, number of lateral roots per cm of primary root, fresh weight (FW), dry weight (DW) and leaf chlorosis of the wild type and 3 independent transgenic lines (*Es*M1, *Es*M2 and *Es*M3), in the absence and presence of 100 mM NaCl. (**a**–**d**), seedlings were grown under standard conditions or (**e**–**h**), seedlings were grown in the presence of 100 mM NaCl. (**a**,**e**) WT, (**b**,**f**) *Es*M1, (**c**,**g**) *Es*M2 and (**d**,**h**) *Es*M3. The seedlings were photographed at 11 days after transfer in standard and under salinity stress conditions. Each grid has 13 mm. (**i**) root length, (**j**) number of lateral roots per cm of primary root, (**k**) fresh weight, (**l**) dry weight and (**m**) leaf chlorosis. Columns represents the mean and the bars the standard error; n = 150 for root length, number of lateral roots per cm of primary root and leaf chlorosis and n = 18 for fresh weight and dry weight. Means and SE with the same letter are not significantly different.

**Figure 2:**
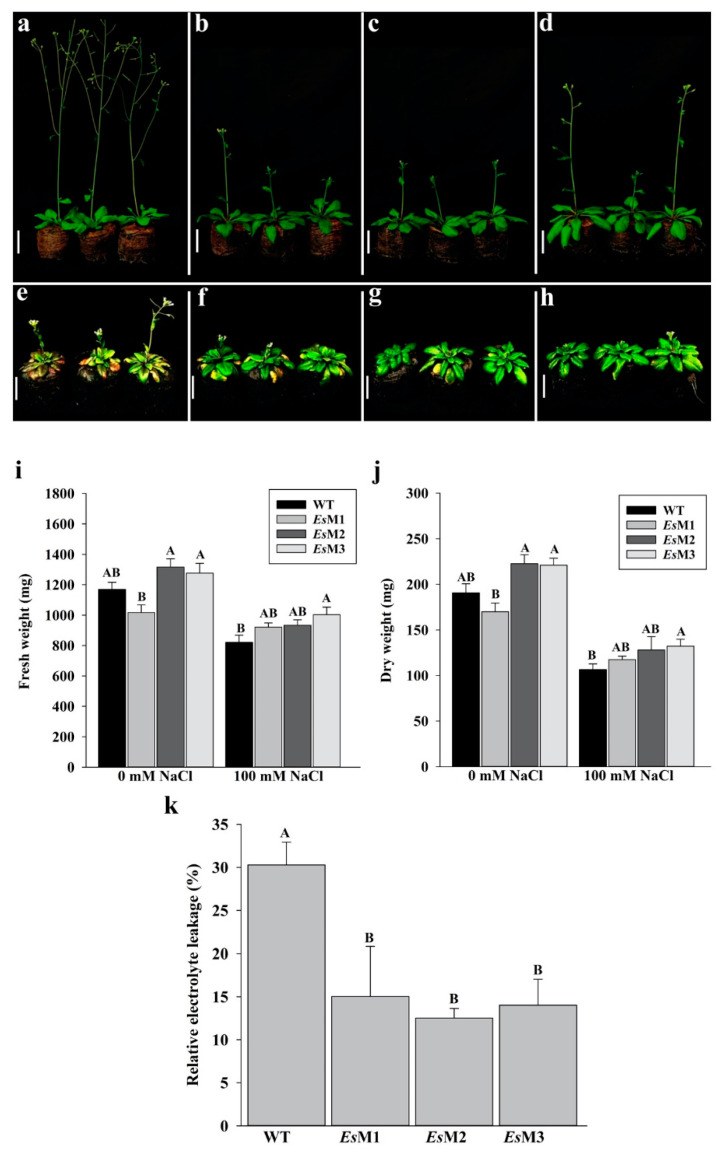
Plants growth, fresh weight (FW), dry weight (DW) and electrolyte leakage of the wild type and 3 independent transgenic lines (*Es*M1, *Es*M2 and *Es*M3), grown in the absence and presence of 100 mM NaCl. Plants (**a**–**d**), were grown under standard conditions while in (**e**–**h**), plants were grown in the presence of 100 mM NaCl. The white bar indicates 3.5 cm. (**a**,**e**) WT, (**b**,**f**) *Es*M1, (**c**,**g**) *Es*M2 and (**d**,**h**) *Es*M3. The plants were photographed at 10 days after irrigation when they were 25 days old. (**i**) fresh weight, (**j**) dry weight and (**k**) electrolyte leakage. Columns represents the mean and the bars the standard error (n = 30). Means and SE with the same letter are not significantly different.

All T5 transgenic seedlings, exhibited improved tolerance to salinity stress when exposed to 100 mM NaCl (Figure 1). Transgenic seedlings subjected to salinity stress showed significantly longer roots, higher number of lateral roots per cm of primary root and reduced leaf chlorosis as compared to the wild type seedlings (Figure 1 and Supplementary Table S3). In standard conditions, no significant differences were recorded in the fresh and dry weight of transgenic lines, as compared to the wild type seedlings, while under salinity stress, the transgenic lines accumulated significantly higher biomass (Figure 1 and Supplementary Table S4). All transgenic lines grew much better than the wild type on peat pellet when subjected to 100 mM NaCl salt stress. After one week of salinity stress, the transgenic lines plants displayed less growth inhibition, had significantly higher fresh and dry weight and less chlorosis compared to the wild type plants (Figure 2 and Supplementary Table S4).

The effect of salinity stress on membrane intactness was estimated by measuring electrolyte leakage. The 15 days old mannitol-producing transgenic plants, exposed to 100 mM NaCl, showed significantly lower electrolyte leakage compared to wild type plants, suggesting higher membrane stability in transgenic lines (Figure 2).

Previous studies have shown that plants tolerant to salinity stress also exhibit tolerance to high temperature stress as both stressors cause *in planta* hyperosmotic conditions, and many of the stress responsive genes are commonly shared ([21]. Indeed, when subjected to high temperature stress seedlings from the T5 generation exhibited improved tolerance compared to the wild type seedlings (Supplementary Figure S2). Transgenic seedlings exposed to high temperature stress (40 °C for 24 h) had significantly higher fresh and dry weight as compared to the wild type plants, while no significant differences were noted in plants grown under standard conditions (Supplementary Figure S2 and Supplementary Table S5).

### 2.3. Transcriptomics of a Mannitol-Producing Line and of Wild Type A. thaliana Grown in Standard Conditions and under Salt Stress

Since we demonstrated that mannitol is produced in transgenic *A. thaliana*, we decided to evaluate to what extent the transcriptome of the transgenic line *Es*M3 is influenced by mannitol, in optimal growth condition and under salt stress. Fifteen days old plants grown on peat pellet were subjected to salinity stress (100 mM NaCl) and leaves were collected 24 and 48 h after treatment. After Illumina sequencing and processing of raw reads, sequences were mapped using TAIR10. A number of 23,387 of *A. thaliana* transcripts were found to be expressed, being present in at least one library from the total number of libraries sequenced. Preliminary global correlation analyses clearly separated the samples (libraries) generated from plants grown in standard conditions from those grown under salt stress (Supplementary Figure S3). Notable differences could also be observed between the 24 h and 48 h time points, after the NaCl treatment. No obvious differences could be observed between the mannitol-producing line and the wild type when plants were grown in standard conditions (Supplementary Figure S4).

**Figure 3:**
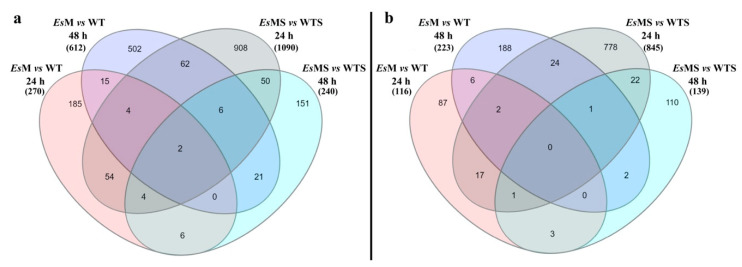
Venn diagram showing the differentially expressed genes (>2-fold up- and down-regulated) in the mannitol transgenic line vs. wild type, in standard (*Es*M and WT, respectively) and salinity stress conditions (*Es*MS and WTS, respectively), at 24 and 48 h. (**a**) genes that were >2-fold up-regulated, (**b**) genes that were >2-fold down-regulated.

Differential gene expression analysis (DGEs) was performed using Cuffdiff software with a cutoff *p* value < 0.05, and a fold change ≥ 2. DGE between mannitol-producing line (*Es*M) and wild type (WT), under standard conditions, identified 270 genes up-regulated at 24 h and 612 genes at 48 h. A smaller number of genes was found to be down-regulated, i.e., 116 and 223 genes at 24 h and 48 h, respectively (Figure 3 and Supplementary Figure S4). These results indicate that mannitol production did not drastically affect overall gene expression in *Es*M vs. WT. This view is supported by the fact that in pairwise comparisons WT 24 h vs. WT 48 h, or in *Es*M 24 h vs. *Es*M 48 h, roughly the same number of genes were found to be differentially expressed in standard conditions (Supplementary Figure S4).

DGE between the mannitol-producing line and wild type, under salt stress conditions, henceforth named *Es*MS and WTS, respectively, identified a large number of genes that were substantially increased at 24 h but not at 48 h. A total number of 1090 of genes were identified to be up-regulated at 24 h in *Es*MS vs. WTS; at 48 h these differences were much smaller, with only 240 genes displaying up-regulation. A similar DGE pattern was observed for genes identified to be down-regulated, with 845 genes and 139 genes at 24 h and 48 h, respectively (Figure 3 and Supplementary Figure S4).

In sharp contrast, the number of genes induced by salinity was quite large, in both the mannitol-producing line and wild type. At 24 h after the salt stress, 1991 genes were up-regulated in WTS vs. WT, and 2731 in *Es*MS vs. *Es*M. At 48 h, the number of genes was further increased in WTS vs. WT while in *Es*MS vs. *Es*M the number of genes (2585) was almost the same as after 24 h. A rather similar number of genes was found to be down-regulated in both the mannitol-producing line and wild type *A. thaliana* (Supplementary Figure S4).

**Figure 4:**
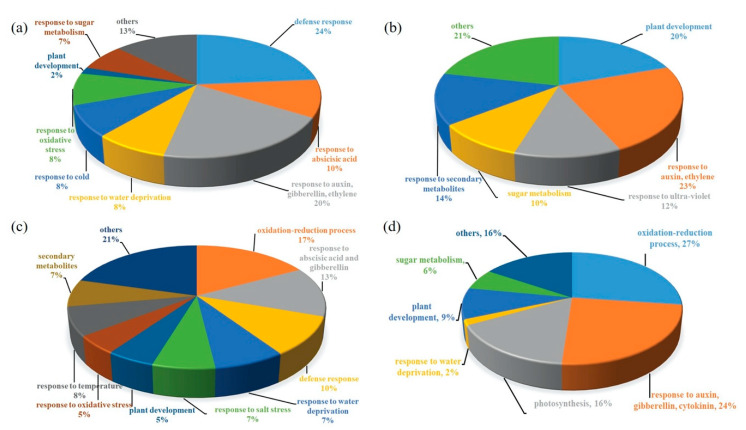
Functional categories grouping of genes which showed >2-fold change in expression, in mannitol-producing line vs. wild type pairwise comparisons, in standard and salinity stress conditions. (**a**) Up-regulated at 24 and 48 h under standard conditions. (**b**) Down-regulated at 24 and 48 h under standard conditions. (**c**) Up-regulated at 24 and 48 h under salinity stress. (**d**) Down-regulated at 24 and 48 h under salinity stress. Percentages for each category represents the number of genes involved in the functions out of total number of genes that were up- and down-regulated >2 fold at *p* value < 0.05 for both 24 h and 48 h time points.

Functional annotation grouping of DGE, in standard conditions, showed that mannitol enhanced expression of a significant number of genes involved in defense mechanisms, responses to hormones such as auxins, gibberellins, ethylene and abscisic acid, oxidative stress and plant development (Figure 4a and Supplementary Dataset S3). Transcript levels of genes involved in categories such as plant development, response to auxin and ethylene, sugar and secondary metabolites and ultraviolet light decreased (Figure 4b and Supplementary Dataset S3).

Salt stress up-regulated several genes involved in oxidation-reduction processes, response to abscisic acid and gibberellin, defense mechanisms, responses to water deprivation, salt stress and temperature and secondary metabolites (Figure 4c and Supplementary Dataset S3). Some of these genes involved in ROS homeostasis, ABA signaling, salinity stress, ion transporters, heat stress, LEA proteins, and other functions, that were significantly up-regulated, and acted in concert, are listed in Supplementary Dataset S2. Several genes that were found to be down-regulated belong to categories that contained genes that were found to be up-regulated, for example, response to auxin, gibberellin, cytokinin, oxidation-reduction processes; however, different categories such as photosynthesis, plant development and sugar metabolism have been also identified (Figure 4d and Supplementary Dataset S3).

To further characterize the effects of mannitol on salinity stress response, transcription factors and genes interconnected within GO:0042538, which comprises all cellular processes related to hyperosmotic salinity stress response, were used to generate a network of co-expressed genes in salinity stress conditions. The relationship among genes was estimated by ATTED-II v. 9.2 (http://atted.jp).

**Figure 5:**
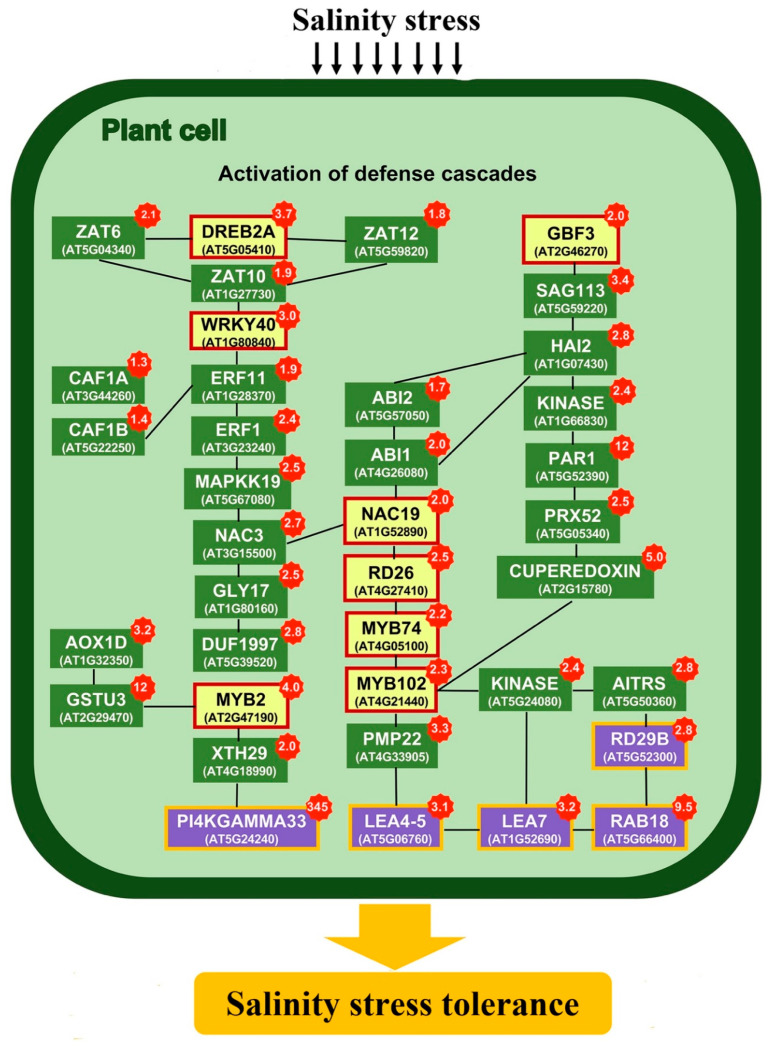
Co-expression analysis of genes induced by salinity stress (GO:0042538). Listed in the yellow box are some of the main transcription factors that regulate the expression of several genes involved in salinity stress tolerance. In green boxes are listed genes involved in the activation of various defense mechanisms. In purple boxes are listed well characterized genes that are up-regulated in salinity and temperature stress in pairwise comparison of *Es*MS vs. WTS. Accession number for these genes and fold increase in expression for both time points are also listed in Supplementary Dataset S3. Values highlighted in red are the fold change of genes, in pairwise comparison of *Es*MS vs. WTS.

Analysis revealed that genes encoding several transcription factors and their target genes in the network, were significantly up-regulated in *Es*MS in contrast to WTS (Figure 5 and Supplementary Dataset S3). For example, one of these transcription factors is *MYB2*, a CaM (calmodulin) binding transcription factor that regulates the expression of stress responsive genes, including that of pyrroline-5-carboxylate synthetase-1 (*P5CS1*), which imparts the salinity stress tolerance by production and accumulation of proline [22]. The expression of *MYB2* (AT2G47190) and *P5CS1* (AT4G28330) increased found to be significantly (4.04 and 2.76 fold, respectively) in *Es*MS vs. WTS (Figure 5 and Supplementary Dataset S3).

**Figure 6:**
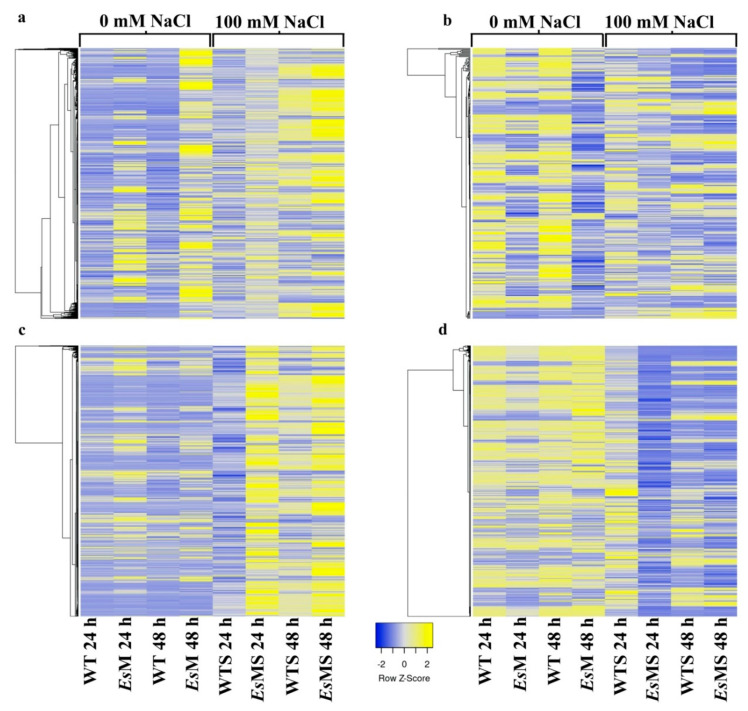
Heat map of selected genes in the mannitol-producing line and wild type, in standard (*Es*M and WT) and salinity stress conditions (*Es*MS and WTS). (**a**) selection based on genes that were up-regulated in *Es*M at 24 and 48 h, under standard conditions (columns 2 and 4). (**b**) selection based on genes that were down-regulated in *Es*M at 24 and 48 h, under standard conditions (columns 2 and 4). (**c**) selection based on genes that were up-regulated in *Es*MS at 24 and 48 h, under salinity stress (columns 6 and 8). (**d**) selection based on genes that were down-regulated in *Es*MS at 24 and 48 h, under salinity stress (columns 6 and 8). Selection of genes was made based on the >2-fold change in expression in *Es*M vs. WT and *Es*MS vs. WTS pairwise comparisons.

Heat map analysis of the differentially expressed genes, in standard conditions, selected based on pairwise *Es*M vs. WT comparison, at 24 h and 48 h, revealed that a large number of genes which were up-regulated in *Es*M (columns 2 and 4) displayed an expression pattern much more similar to that of plants subjected to salinity stress, i.e., to that of *Es*MS (columns 6 and 8) and WTS (columns 5 and 7), as compared to WT (Figure 6a,b). This trend was even more noticeable when the expression patterns of *Es*MS and WTS were compared 24 h after the salt stress (columns 5 and 6, respectively) and 48 h (columns 7 and 8, respectively). At 24 h the expression patterns of *Es*MS and WTS the were clearly less similar compared to 48 h (a). This trend was less noticeable for genes that were down regulated in *Es*M, at 24 h and 48 h (Figure 6b). However, when comparing the expression pattern of the genes found to be differentially up-regulated or down-regulated, at 24 h and 48 h, in salinity stress, the same trend of delayed changes in gene expression at 24 h in WTS, as compared to *Es*MS, was clearly detected (Figure 6c,d).

At 24 h, the expression pattern of WTS (column 5) was found to be totally different from that of *Es*MS (column 6) while at 48 h the expression patterns of *Es*MS and WTS showed quite a few similarities (WTS, column 7; *Es*MS, column 8) (Figure 6c,d). Overall, because the expression patterns in WTS and *Es*MS tend to be more alike at 48 h after salt stress and because the trend is driven by the expression pattern of *Es*MS and not that of WTS these results suggest a faster response to salt stress in the mannitol-producing line as compared to the wild type *A. thaliana*.

Multiple comparisons among time points and conditions identified two genes that showed ≥ 2 fold up-regulation across time points and conditions: *AtPI4KGAMMA3* (AT5G24240), and *QQS* (*Qua-Quine Starch*; AT3G30720). *AtPI4KGAMMA3* was found to be >100 fold up-regulated while of *QQS* > 3.3 fold up-regulated in standard and salt stress conditions, in all time points; the expression of these genes was not strongly influenced by salinity stress (Supplementary Dataset S1). *ATPI4KGAMMA3* encodes a type II phosphoinositide 4-kinase that is involved in the response to abscisic acid and salt stress as well as in the regulation of flower development [23]. In *A. thaliana*, *QQS* was shown to modulate carbon/nitrogen allocation by increasing the protein content, and decreasing the total starch content [24,25]. Starch degradation is positively corelated with increased abiotic stress tolerance and is associated with the release of oligosaccharides that function as osmolytes, as precursors for other intermediates necessary for energy production or signaling molecules [26,27,28]. The expression of several α-amylases and β-amylase, known to involved in starch degradation [29], was found to be significantly up-regulated in *Es*MS vs. WTS.

## 3. Discussion

Studies carried out over the past 25 years, by expressing *E. coli mtlD* or celery *M6PR* in a variety of plants, showed improvement in the tolerance of transgenic plants to salt stress. These results were obtained in spite of the fact that mannitol concentrations in the transgenic plants were significantly lower than in the plants in which this sugar alcohol acts naturally as an osmolyte [3,11,12,13,14]. As a single gene was cloned (*E. coli mtlD* or celery *M6PR*), while the conversion to mannitol requires another dephosphorylation step, all previous studies proposed the presence of a putative endogenous phosphatase that converts M1P to mannitol [5,11,12,13,14]. In this study, the genes *EsM1PHD1* and *EsM1Pase2* [19,20] from the brown algal model *Ectocarpus* sp., were used to produce mannitol in *A. thaliana*. The amount of mannitol was lower than that reported in previous studies [3,5,6,11,12,13,14] suggesting that in *A. thaliana EsM1PHD1* is less efficient compared to *E. coli mtlD* or celery *M6PR.* Nevertheless, the three transgenic lines characterized phenotypically exhibited improved tolerance to salinity and temperature stress. These results, and those reported earlier, suggest that specific networks or pathways are activated by low concentrations of mannitol leading to enhanced abiotic stress tolerance. A microarray study carried out by Chan, Grumet and Loescher [6], at 6 days after the salinity stress (100 mM NaCl), using *A. thaliana* expressing celery *M6PR,* found that improved salinity stress tolerance was likely due to differential expression of a variety of genes involved in processes such as salinity and dehydration stress, oxidoreductive processes, sugar and abscisic acid (ABA) metabolism, and cell wall synthesis.

To our knowledge, no other comprehensive studies, including transcriptomics and/or metabolomics, have been carried out to analyze the response to salinity stress at more than one time point, and early during the stress, in any other mannitol-producing transgenic plant. The complexity of the response at early time points is crucial, as it can reveal if plants are primed for long term survival in osmotic stress and water deficit conditions. In the current study transcriptomics data was generated at early time points (24 h and 48 h) after exposure to 100 mM salinity stress.

Our study identified hundreds of genes that were up- and down-regulated in the mannitol overexpression line compared to wild type *A. thaliana* in standard conditions while two to three thousand genes were found to be differentially expressed in salt stress. Heat map analyses suggested that low amounts of mannitol might prime the response of plants to salt stress, as under salt stress conditions the expression pattern of WTS was quite different from that of *Es*MS at 24 h but was much more similar at 48 h. This proposition is also supported by the fact that a much larger number of genes were found to be differentially expressed at 24 h (1090 up- and 845 down-regulated) compared to 48 h (240 up- and 139 down-regulated) in *Es*MS vs. WTS. In addition, after 24 h of salinity stress, the total number of differentially expressed genes in mannitol-producing line (*Es*MS vs. *E*sM) was higher (~2700) than in the wild type (WTS vs. WT) (<2000); notably, after 48 h, the number of differentially expressed genes was comparable.

Functional analysis showed that various categories of genes are differentially expressed, and these functions cover a wider range than previously suggested, including hormonal regulation, defense mechanisms, plant development, photosynthesis, and secondary metabolism. These categories comprise a plethora of genes known to be involved in various abiotic stress, including a large number of crucial transcription factors, K^+^ homeostasis under salinity conditions, ROS signaling and radical scavengers and ABA signaling. When the results of the current study were compared to the findings of Chan et al. [6] several notable differences became evident. In our study, under standard conditions, a much lower number of genes were differentially expressed in the mannitol-producing line compared to the wild type. The number of genes found to be >2 fold up-regulated varied between 270 and 612 and those down-regulated between 116 and 223, at 24 h and 48 h, respectively. In contrast, Chanet al. [6] reported 1204 genes to be up-regulated and 1068 down-regulated. In salt stress, over 2000 genes were found to be up- or down-regulated, at both time points, while Chan et al. [6] reported a much lower number of differentially regulated genes (277 up-regulated and 487 down-regulated genes). These contrasting results might be due to differences in the transcriptomics approach, that is, microarrays vs. RNA-seq, time points (6 days vs. 24 h and 48 h), and the quantity of mannitol produced (up to 6 µmol g^−1^ fresh weight vs. 0.042 µmol g^−1^ fresh weight), when comparing Chan et al. [6] and the current study, respectively.

### 3.1. Stress Responsive Genes

Transgenic plants overexpressing *AtNCED3* have higher amount of ABA and were reported to be tolerant to water stress conditions [30]. The expression of *NCED3* (AT3G14440) was significantly higher (3.86 fold) in *Es*MS vs. WTS. Similarly, the expression of *RD29A*, *KIN1*, *RD17*, *COR15A* and *ERD10*, that are targets of the salt stress inducible transcription factor DREB1A was found to be significantly higher in *Es*MS vs. WTS. The expression of the related genes *RD29B* (AT5G52300) and *RAB18* (AT5G66400) was also found to be significantly higher at 24 h (2.81 and 9.51 fold respectively) and at 48 h (1.50- and 2.33-fold, respectively) in *Es*MS vs. WTS (Figure 5 and Supplementary Dataset S3).

The expression of several heat shock responsive genes was significantly up-regulated in the transgenic line as compared to wild type plant. For instance, *HSP70* (AT3G12580) which has been shown to confer higher tolerance to heat, salinity and drought stress [31] is also one of the genes that was found to be up-regulated (2.1 fold) in *Es*MS vs. WTS.

Role of LEA proteins in abiotic stress tolerance is well known [32,33,34]. *ERD5* and 6 encode a proline dehydrogenase and a sugar transporter, respectively [33]. The expression of these genes was significantly up-regulated in *Es*M line under salinity stress (Figure 5 and Supplementary Dataset S3). Among the different LEA proteins, the early responsive to dehydration (*ERD*) *10* and *14* belongs to the group 2 LEAs, also known as dehydrin (DHN) family proteins. DHNs play an important role in ion sequestration, membrane stabilization and acts as the chaperons [35]. The expression of *ERD10* (AT1G20450) and *ERD14* (AT1G76180) was found to be significantly up-regulated (4.50 and 2.16 fold, respectively) in *Es*MS vs. WTS.

### 3.2. K^+^ Homeostasis Under Salinity Stress

Higher concentration of NaCl outside the roots leads to an influx of calcium; its increased cytosolic concentration causes the activation of stress responsive pathways. Lee, et al. [36], showed that ANN1 is a plasma membrane protein whose expression is triggered by hydroxyl radical (HO^°^) (^•^HO). The expression of ANN1 (AT1G35720) was significantly up-regulated (3.70-fold) in *Es*MS vs. WTS. Accumulation of ANN1 in root has been demonstrated as a negative regulation of Na^+^ influx [37]. Moreover, it reduces the activity of plasma membrane localized guard cell outwardly rectifying K^+^ channel (GORK), [38], therefore playing a role ion homeostasis by preventing the influx of Na^+^ in root and efflux of K^+^ [39].

In plants, Na^+^ and K^+^/H^+^ antiporters play an important role to maintain ion homeostasis. Na^+^/H^+^ antiporters localized at plasma membrane are involved in Na^+^ efflux [40] whereas when localized at tonoplast they are involved in Na^+^ compartmentalization into vacuoles [41]. In *A. thaliana NHX1* (AT5G27150) has been shown to sequester Na^+^ into vacuoles [42], and the expression of this gene was found to be significantly up-regulated (2.15-fold) in *Es*MS vs. WTS. *NHX1* was also shown to mediate K^+^ transport thus playing a role in K^+^ homeostasis [41].

The fact that K^+^ homeostasis might be slightly different in the *Es*MS when compared to WTS is supported by the fact that the expression of *GORK* (AT5G37500) and *SKOR* (AT3G02850) was significantly higher (3.03- and 2.85-fold, respectively) in *Es*MS vs. WTS. In *A. thaliana* these genes encode shaker-like outward-rectifying K^+^ channels which play important roles in K^+^ hemostasis during the challenging environmental conditions [43]. Also, the expression of *KUP6* (AT1G70300) and *KAT1* (AT5G46240), which encode K^+^ uptake permeases [44] was significantly up-regulated (2.68- and 2.21-fold, respectively) in *Es*MS vs. WTS.

### 3.3. ROS Signaling and Radical Scavengers

ROS have important roles in signaling; however, to act as a signaling molecule it has to be maintained at steady state levels, otherwise it can cause detrimental effects to the cells [45]. To maintain the steady state levels plant evolved scavenging mechanisms which involve enzymes such as superoxide dismutases (*SOD*), catalases (*CAT*) and ascorbate peroxidases (*APX*) [46]. In response to abiotic stresses the level of ROS can also be reduced by the increased activity of alternative oxidases (*AOXs*). The expression of *AOX1D* (AT1G32350) was found to be significantly higher at 24 and 48 h (3.20- and 5.48-fold, respectively) in *Es*MS as compared to WTS (Figure 5 and Supplementary Dataset S3). In plants, ascorbate peroxidases are responsible for the conversion of H_2_O_2_ into water by oxidizing ascorbate, which is regenerated by mono-dehydroascorbate (*MDHAR*) and de-hydroascorbate reductases (*DHAR*). Increased activity of *MDHARs* has been shown to provide protection against oxidative stress. *DHAR1* (AT1G19570) and *DHAR2* (AT1G75270) were found to be significantly up-regulated (3.09- and 2.07-fold, respectively) while *MDHAR* (AT3G09940) was expressed only in *Es*MS vs. WTS. Moreover, the existence of an ascorbate independent pathway suggests that other antioxidants including glutathione peroxidases (*GPXs*) and glutathione-s-transferases (*GSTs*) can reduce the H_2_O_2_ and other hydroperoxide radicals without the expense of ascorbate [47]. In the current study, DGE analysis revealed that some of the genes related to glutathione-s-transferase and peroxidase family were significantly up-regulated in *Es*MS vs. WTS. *APX2* (ascorbate peroxidase 2) was shown to improve water use in water deficit conditions [48]. The expression of *APX2* (AT3G09640) was found to be 18.5-fold up-regulated in *Es*MS as compared to WTS. The higher expression of these genes and of other genes with similar functions in *Es*MS suggest that mannitol production might be associated with improved ROS detoxification and higher water use efficiency, which can lead to improved tolerance to high salinity and temperature stress. Glyoxalase has been suggested as the direct target of *RD26*, participating in ROS scavenging [49]. The expression of *GSTs* family proteins and *RD26* was found to be significantly up-regulated in *Es*MS, indicating another possible mechanism involved in the improved stress tolerance observed in this study.

### 3.4. ABA Signaling

In *A. thaliana*, 2C protein phosphatases (PP2Cs) clade A type have been demonstrated to act as negative regulators of ABA signaling [50]. These PP2Cs (*ABI1, ABI2, HAI1, HAI2*) interact with *SnRKs* (in particular 2.2, 2.3 and 2.6), which are key players in ABA signaling, and inhibit them by dephosphorylation [51]. The expression of *ABI1* (AT4G26080), *ABI2* (AT5G57050), *HAI1* (AT5G59220), *HAI2* (AT1G07430) and *HAI3* (AT2G29380) was significantly up-regulated (2.41-, 2.13-, 3.39-, 2.74- and 4.02-fold, respectively) in *Es*MS vs. WTS. However, during ABA induction the ABA receptors pyrabactin resistance-like (*PYL*) bind to ABA, suppressing the activity of PP2Cs. This activates the *SnRK2s* which get autophosphorylated and then phosphorylate the downstream proteins that are involved in abiotic stress responses [52]. The expression of *SnRK 2.2* (AT3G50500), *SnRK2.3* (AT5G66880), and *SnRK 2.6* (AT4G33950) was significantly higher (2.14-, 1.40- and 1.68-fold, respectively) in *Es*MS vs. WTS. The expression of ABA receptors *PYL2* (AT2G26040), *PYL5* (AT5G05440) and *PYL7* (AT4G01026) was also significantly higher (2.45-, 1.87- and 2.05-fold, respectively) in *Es*MS vs. WTS. These results suggest that low amounts of mannitol altered the ABA signaling, and thus, the expression of the downstream stress responsive genes.

## 4. Materials and Methods

### 4.1. Cloning of Ectocarpus sp. M1PDH1and M1Pase2 Genes and Transformation of A. thaliana

*Ectocarpus* sp. *M1Pase2* and codon-optimized *M1PDH1* [19], under separate 35S promoters and OCS terminators, were cloned in pEarleyGate 100 obtained from ABRC (Columbus, OH, USA) using the multisite Gateway Pro Four Fragment recombination technology (Gateway^®^ Technology Multisite, Invitrogen, Mississauga, ON, Canada). The expression plasmid containing both genes was transformed into *Agrobacterium* strain GV310 (pMB90) and then *A. thaliana* (L.) Heynh, ecotype Columbia (Col-0) (Lehle Seeds, Round Rock, TX, USA) was used to generate transgenic plants, using the floral dip method [53].

### 4.2. Selection of Transformants and Homozygosity

Positive transformants were selected by seed germination and seedling growth on plates containing half strength Murashige and Skoog medium supplemented with 35 µg/mL ammonium glufosinate (Sigma, Mississauga, ON, Canada). Three independent transgenic lines were selected, and seeds collected from the fifth generation were used for further experiments. Transgene expression was verified by qPCR as described below.

### 4.3. Salinity Stress Tolerance

For *in vitro* experiments seeds were germinated in half strength MS medium, supplemented with 1% (*w*/*v*) sucrose. Seedlings were maintained at 22 °C, in a 16-h light/8-h dark cycle. After 4 ½ days of growth, uniform seedlings were transferred on plates containing the same medium, in the absence or presence of 100 mM NaCl, and grown in the same conditions as mentioned before. Root length was marked on the day of transfer; after 7 days, leaf chlorosis was recorded, and plates were scanned with a high-resolution scanner (Expression 10,000 XL, Epson, Markham, ON, Canada). Root length was measured with Image J software (Research Services Branch, NIH, Bethesda, MD, USA). Fresh weight of seedlings was recorded 11 days after the transfer, then samples were dried. Experiments were repeated three times with 60 seedlings per transgenic line per treatment. Plants grown in these conditions have been referred to as seedlings throughout this paper.

In vivo experiments were carried out using Jiffy peat pellets (Jiffy, Shippagan, NB, Canada). Experiments were started using 15 days old plants. Peat pellets were irrigated once with 40 mL of 200 mM NaCl per plant, giving a final concentration of NaCl in the pellet of 100 mM. After 5 days, 20 mL of water was added to each plant; subsequent watering was done at 4 days interval, for 3 weeks. After 3 weeks plants biomass was recorded. Experiments were repeated three times with 10 plants per transgenic line per treatment. Plants grown in these conditions have been referred to as adult plants throughout this paper. Data from all experiments were analyzed using ANOVA followed by Tukey’s at an α < 0.05 in SAS 9.1.2 (2001, SAS Institute, Cary, NC, USA).

### 4.4. Heat Stress Tolerance

Seedlings were generated on plates as described earlier. Ten days old seedlings were exposed to 40 °C for 24 h. Seedlings were then grown under standard conditions and biomass was recorded at 8 days after the temperature stress. Experiments were repeated three times with 60 seedlings per transgenic line per treatment.

### 4.5. Electrolyte Leakage

Plants were grown on peat pellets as described earlier. Fifteen days old plants were irrigated with 40 mL of 100 mM NaCl per plant and after 24 h the leaves were harvested and placed in scintillation vials containing 20 mL of deionized water. The experiment was repeated two times and each experiment had three biological replicates, each represented by pooled leaves from three plants. Electrolyte leakage was recorded using SympHony SB70C (VWR, Mississauga, ON, Canada) conductivity meter.

### 4.6. Quantification of Mannitol Levels in Transgenic Lines by LC-MS

Leaves from 18-day-old plants, grown on peat pellets, were harvested, frozen in liquid nitrogen, and then lyophilized. Approximately 5 mg of freeze-dried leaves were mixed with 1 mL cold 90% methanol and ribitol (internal standard, Sigma-Aldrich, St. Louis, MO, USA). Samples were dried, cleaned up by suspending in 500 µL of acetonitrile/water (1:1), dried again and then resolubilized in 500 µL methanol for LC-MS analysis.

The LC-MS system used for mannitol quantification included an UltiMate 3000 LC pump (Thermo Fisher Scientific, Waltham, MA, USA) coupled to an Exactive™ high resolution mass spectrometer (Thermo Fisher Scientific), equipped with an electrospray ionization source. Separation was carried out on an Acquity BEH Amide column (2.1 × 100 mm 1.7 µm, Waters, Milford, MA, USA) Peak area based on the accurate mass measurement of mannitol at m/z 181.07176 was used to determine its concentrations in the plant tissue samples. Two extraction replicates and two analytical replicates from three biological samples were analysed for each of the transgenic lines.

### 4.7. Plant Samples, RNA Isolation, Library Construction and Next Generation Sequencing

Plants were grown on peat pellet and 15 days old plants were exposed to salt stress as described above. Leaf tissues were collected 24 and 48 h after the exposure to salinity stress and stored in RNA Later solution (Life Technologies, Mississauga, ON, Canada). Leaf samples were ground in liquid nitrogen and RNA isolation was performed using the E.Z.N.A. Plant RNA Kit (Omega Bio-Tek, Shanghai, China). Library construction and sequencing using the Illumina HiSeq2500 technology were done at Beijing Novogene Bioinformatics Technology Co. Ltd. (Beijing, China). A total of 68.03 GB data were generated and the clean data was 66.35 GB. The 24 sequenced libraries were from the wild type and from transgenic line *Es*M3. The experiment had two time points (24 and 48 h after salt stress), in standard conditions (i.e., no salt, samples described as WT and EsM) and in presence of salt stress (samples described as WTS and EsMS); each time point and condition had three biological replicates.

### 4.8. Next Generation Sequencing Data Processing

Sequence trimming (adapters and low-quality reads) was performed by Beijing Novogene Bioinformatics Technology Co. Ltd. Mapping to *A. thaliana* genome was done using TAIR10 (ftp://ftp.arabidopsis.org/home/tair) and TopHat2 software with default parameters. Differential gene expression was performed using Cufflinks, and identification of DEGs was performed using Cuffdiff software with a cutoff *p* value < 0.05, and a fold change ≥ 2. Functional annotation was done using DAVID Bioinformatics Resources 6.8 website (https://david.ncifcrf.gov). Venn diagrams [54] were produced from pairwise and multiple comparisons. Heat maps were generated using Heatmapper [55] at http://www.heatmapper.ca; data was analyzed by clustering (average linkage) using the Euclidean distance measurement method. Regulatory networks related to hyperosmotic and salinity stress were generated based on the information available from ATTED-II v. 9.2 (http://atted.jp). Transcriptomics data was submitted to sequence read archive (SRA); submission ID: SUB8462528 and project ID: PRJNA674912.

### 4.9. Real-Time Quantitative PCR

Transgenic lines were analyzed by RT-qPCR to assess the expression of *Ectocarpus* sp. *M1PDH1* and *M1Pase2*. Total RNA was extracted using GeneJET plant RNA purification kit (Thermo Scientific, Mississauga, ON, Canada). DNAse treated RNA was converted into cDNA using the RevetAID cDNA Synthesis kit (Thermo Scientific). The relative transcript levels were determined using the gene specific primers and actin 2 (AT3G18780) primers (Supplementary Table S1) as the internal control on the StepOne plus Real-Time PCR system (Applied Biosystems, Mississauga, ON, Canada), using iTaq SYBR Green (Bio-Rad, Mississauga, ON, Canada). Transcript abundance was determined using the comparative C_T_ method for relative quantification by employing the samples from the time point and the tissue with the lowest gene expression as calibrators.

## 5. Conclusions

The expression, for the first time, of the two genes involved in mannitol production, from the brown alga *Ectocarpus* sp. in *A. thaliana* resulted in improved tolerance to salinity and temperature stress though mannitol concentrations were not higher than in the plants in which *E.coli mtlD* or celery *M6PR* genes were used to drive mannitol production. Transcriptomics analysis of a mannitol-producing line and of the wild type, grown in standard conditions and under salt stress, revealed a large number of genes that are differentially up- and down-regulated in the mannitol-producing transgenic line. These genes cover a wide range of functions, that were not limited to salt and oxidative stress responses, as previous studies suggested. These findings unravel new facets of the complex mechanisms through which low concentrations of mannitol improve plant’s tolerance to salinity stress.

## Supplementary Materials

Supplementary materials can be found at https://www.mdpi.com/2223-7747/9/11/1508/s1. All datasets generated for this study are included in the article/Supplementary Material. The Supplementary Material for this article can be found online at: Supplementary Dataset S1. List of the genes identified in the transcriptomics data. WT-wild type, *Es*M-mannitol producing line, WTS-wild type under salinity stress, *Es*MS-mannitol producing line under salinity stress. Tair10 and Araport11 are Arabidopsis information resources database and fragments per kilobase of transcripts per million (FPKM) are expression values. Supplementary Dataset S2. List of selected up-regulated genes in *Es*MS vs. WTS grouped in different functional categories. Supplementary Dataset S3. Functional categories grouping of genes which showed >2-fold change in expression at *p* value < 0.05, in mannitol vs. wild type pairwise comparisons, in standard and salinity stress conditions. Data from this dataset was used to generate Figure 4.

## References

[1] Zhu J.-K. (2016). Abiotic stress signaling and responses in plants. Cell.

[2] Verslues P.E., Agarwal M., Katiyar-Agarwal S., Zhu J., Zhu J.K. (2006). Methods and concepts in quantifying resistance to drought, salt and freezing, abiotic stresses that affect plant water status. Plant J..

[3] Patel T.K., Williamson J.D. (2016). Mannitol in plants, fungi, and plant-fungal Interactions. Trends Plant Sci..

[4] Tonon T., Li Y., McQueen-Mason S. (2017). Mannitol biosynthesis in algae: More widespread and diverse than previously thought. New Phytol..

[5] Abebe T., Guenzi A.C., Martin B., Cushman J.C. (2003). Tolerance of mannitol-accumulating transgenic wheat to water stress and salinity. Plant Physiol..

[6] Chan Z., Grumet R., Loescher W. (2011). Global gene expression analysis of transgenic, mannitol-producing, and salt-tolerant *Arabidopsis thaliana* indicates widespread changes in abiotic and biotic stress-related genes. J. Exp. Bot..

[7] Chiang Y.-J., Stushnoff C., McSay A., Jones M.L., Bohnert H. (2005). Overexpression of mannitol-1-phosphate dehydrogenase increases mannitol accumulation and adds protection against chilling injury in *Petunia*. J. Am. Soc. Hortic. Sci..

[8] Patel T., Krasnyanski S., Allen G., Panthee D., Louws F., Williamson J. (2015). Tomato plants overexpressing a celery mannitol dehydrogenase (*mtd*) have decreased susceptibility to *Botrytis cinerea*. Am. J. Plant Sci..

[9] Prabhavathi V., Rajam M.V. (2007). Mannitol-accumulating transgenic eggplants exhibit enhanced resistance to fungal wilts. Plant Sci..

[10] Sickler C.M., Edwards G.E., Kiirats O., Gao Z., Loescher W. (2007). Response of mannitol-producing Arabidopsis thaliana to abiotic stress. Funct. Plant Biol..

[11] Tarczynski M.C., Jensen R.G., Bohnert H.J. (1992). Expression of a bacterial *mtlD* gene in transgenic tobacco leads to production and accumulation of mannitol. Proc. Natl. Acad. Sci. USA.

[12] Tarczynski M.C., Jensen R.G., Bohnert H.J. (1993). Stress protection of transgenic tobacco by production of the osmolyte mannitol. Science.

[13] Zhifang G., Loescher W. (2003). Expression of a celery mannose 6-phosphate reductase in *Arabidopsis thaliana* enhances salt tolerance and induces biosynthesis of both mannitol and a glucosyl-mannitol dimer. Plant Cell Environ..

[14] Thomas J.C., Sepahi M., Arentall B., Bohnert H.J. (1995). Enhancement of seed germination in high salinity by engineering mannitol expression in *Arabidopsis thaliana*. Plant Cell Environ..

[15] Coelho S.M., Scornet D., Rousvoal S., Peters N.T., Dartevelle L., Peters A.F., Cock J.M. (2012). *Ectocarpus*: A Model Organism for the Brown Algae. Cold Spring Harb. Protoc..

[16] Black W.A.P. (1950). The seasonal variation in weight and chemical composition of the common British *Laminariaceae*. J. Mar. Biol. Assoc. U. K..

[17] Montecinos A.E., Couceiro L., Peters A.F., Desrut A., Valero M., Guillemin M.-L. (2017). Species delimitation and phylogeographic analyses in the *Ectocarpus* subgroup siliculosi (Ectocarpales, Phaeophyceae). J. Phycol..

[18] Gravot A., Dittami S.M., Rousvoal S., Lugan R., Eggert A., Collén J., Boyen C., Bouchereau A., Tonon T. (2010). Diurnal oscillations of metabolite abundances and gene analysis provide new insights into central metabolic processes of the brown alga *Ectocarpus siliculosus*. New Phytol..

[19] Bonin P., Groisillier A., Raimbault A., Guibert A., Boyen C., Tonon T. (2015). Molecular and biochemical characterization of mannitol-1-phosphate dehydrogenase from the model brown alga *Ectocarpus* sp.. Phytochemistry.

[20] Groisillier A., Shao Z., Michel G., Goulitquer S., Bonin P., Krahulec S., Nidetzky B., Duan D., Boyen C., Tonon T. (2014). Mannitol metabolism in brown algae involves a new phosphatase family. J. Exp. Bot..

[21] Sakuma Y., Maruyama K., Qin F., Osakabe Y., Shinozaki K., Yamaguchi-Shinozaki K. (2006). Dual function of an Arabidopsis transcription factor *DREB2A* in water-stress-responsive and heat-stress-responsive gene expression. Proc. Natl. Acad. Sci. USA.

[22] Yoo J.H., Park C.Y., Kim J.C., Do Heo W., Cheong M.S., Park H.C., Kim M.C., Moon B.C., Choi M.S., Kang Y.H. (2005). Direct interaction of a divergent CaM isoform and the transcription factor, MYB2, enhances salt tolerance in Arabidopsis. J. Biol. Chem..

[23] Akhter S., Uddin M.N., Jeong I.S., Kim D.W., Liu X.M., Bahk J.D. (2016). Role of Arabidopsis *At PI 4Kγ3,* a type II phosphoinositide 4-kinase, in abiotic stress responses and floral transition. Plant Biotechnol. J..

[24] Li L., Zheng W., Zhu Y., Ye H., Tang B., Arendsee Z.W., Jones D., Li R., Ortiz D., Zhao X. (2015). *QQS* orphan gene regulates carbon and nitrogen partitioning across species via NF-YC interactions. Proc. Natl. Acad. Sci. USA.

[25] Qi M., Zheng W., Zhao X., Hohenstein J.D., Kandel Y., O’Conner S., Wang Y., Du C., Nettleton D., MacIntosh G.C. (2019). QQS orphan gene and its interactor NF-YC4 reduce susceptibility to pathogens and pests. Plant Biotechnol. J..

[26] Thalmann M., Santelia D. (2017). Starch as a determinant of plant fitness under abiotic stress. New Phytol..

[27] Rook F., Hadingham S.A., Li Y., Bevan M.W. (2006). Sugar and ABA response pathways and the control of gene expression. Plant Cell Environ..

[28] Kempa S., Krasensky J., Dal Santo S., Kopka J., Jonak C. (2008). A central role of abscisic acid in stress-regulated carbohydrate metabolism. PLoS ONE.

[29] Lloyd J.R., Kossmann J., Ritte G. (2005). Leaf starch degradation comes out of the shadows. Trends Plant Sci..

[30] Qin X., Zeevaart J.A. (2002). Overexpression of a *9-cis-epoxycarotenoid dioxygenase* gene in *Nicotiana plumbaginifolia* increases abscisic acid and phaseic acid levels and enhances drought tolerance. Plant Physiol..

[31] Wang W., Vinocur B., Shoseyov O., Altman A. (2004). Role of plant heat-shock proteins and molecular chaperones in the abiotic stress response. Trends Plant Sci..

[32] Hundertmark M., Hincha D.K. (2008). LEA (late embryogenesis abundant) proteins and their encoding genes in *Arabidopsis thaliana*. BMC Genomics.

[33] Kiyosue T., Abe H., Yamaguchi-Shinozaki K., Shinozaki K. (1998). *ERD6*, a cDNA clone for an early dehydration-induced gene of Arabidopsis, encodes a putative sugar transporter. BBA Biomembr..

[34] Jia F., Qi S., Li H., Liu P., Li P., Wu C., Zheng C., Huang J. (2014). Overexpression of Late Embryogenesis Abundant 14 enhances Arabidopsis salt stress tolerance. Biochem. Biophys. Res. Commun..

[35] Tunnacliffe A., Wise M.J. (2007). The continuing conundrum of the LEA proteins. Naturwissenschaften.

[36] Lee S., Lee E.J., Yang E.J., Lee J.E., Park A.R., Song W.H., Park O.K. (2004). Proteomic identification of annexins, calcium-dependent membrane binding proteins that mediate osmotic stress and abscisic acid signal transduction in Arabidopsis. Plant Cell.

[37] Guo K.M., Babourina O., Christopher D.A., Borsics T., Rengel Z. (2008). The cyclic nucleotide-gated channel, AtCNGC10, influences salt tolerance in Arabidopsis. Physiol. Plant..

[38] Demidchik V., Cuin T.A., Svistunenko D., Smith S.J., Miller A.J., Shabala S., Sokolik A., Yurin V. (2010). Arabidopsis root K+-efflux conductance activated by hydroxyl radicals: Single-channel properties, genetic basis and involvement in stress-induced cell death. J. Cell Sci..

[39] Laohavisit A., Richards S.L., Shabala L., Chen C., Colaço R.D., Swarbreck S.M., Shaw E., Dark A., Shabala S., Shang Z. (2013). Salinity-induced calcium signaling and root adaptation in Arabidopsis require the calcium regulatory protein annexin1. Plant Physiol..

[40] Qiu Q.-S., Barkla B.J., Vera-Estrella R., Zhu J.-K., Schumaker K.S. (2003). Na+/H+ exchange activity in the plasma membrane of Arabidopsis. Plant Physiol..

[41] Apse M.P., Sottosanto J.B., Blumwald E. (2003). Vacuolar cation/H+ exchange, ion homeostasis, and leaf development are altered in a T-DNA insertional mutant of *AtNHX1*, the Arabidopsis vacuolar Na+/H+ antiporter. Plant J..

[42] Zhang H.-X., Blumwald E. (2001). Transgenic salt-tolerant tomato plants accumulate salt in foliage but not in fruit. Nat. Biotechnol..

[43] Pilot G., Gaymard F., Mouline K., Chérel I., Sentenac H. (2003). Regulated expression of Arabidopsis Shaker K+ channel genes involved in K+ uptake and distribution in the plant. Plant Mol. Biol..

[44] Osakabe Y., Arinaga N., Umezawa T., Katsura S., Nagamachi K., Tanaka H., Ohiraki H., Yamada K., Seo S.-U., Abo M. (2013). Osmotic stress responses and plant growth controlled by potassium transporters in Arabidopsis. Plant Cell.

[45] Mittler R. (2002). Oxidative stress, antioxidants and stress tolerance. Trends Plant Sci..

[46] Willekens H., Chamnongpol S., Davey M., Schraudner M., Langebartels C., Van Montagu M., Inzé D., Van Camp W. (1997). Catalase is a sink for H2O2 and is indispensable for stress defence in C3 plants. EMBO J..

[47] Chang C.C., Ślesak I., Jordá L., Sotnikov A., Melzer M., Miszalski Z., Mullineaux P.M., Parker J.E., Karpińska B., Karpiński S. (2009). Arabidopsis chloroplastic glutathione peroxidases play a role in cross talk between photooxidative stress and immune responses. Plant Physiol..

[48] Rossel J.B., Walter P.B., Hendrickson L., Chow W.S., Poole A., Mullineaux P.M., Pogson B.J. (2006). A mutation affecting *ASCORBATE PEROXIDASE 2* gene expression reveals a link between responses to high light and drought tolerance. Plant Cell Environ..

[49] Dixon D.P., Cummins I., Cole D.J., Edwards R. (1998). Glutathione-mediated detoxification systems in plants. Curr. Opin. Plant Biol..

[50] Antoni R., Gonzalez-Guzman M., Rodriguez L., Rodrigues A., Pizzio G.A., Rodriguez P.L. (2012). Selective inhibition of clade A phosphatases type 2C by PYR/PYL/RCAR abscisic acid receptors. Plant Physiol..

[51] Fujii H., Chinnusamy V., Rodrigues A., Rubio S., Antoni R., Park S.-Y., Cutler S.R., Sheen J., Rodriguez P.L., Zhu J.-K. (2009). In vitro reconstitution of an abscisic acid signalling pathway. Nature.

[52] Soon F.-F., Ng L.-M., Zhou X.E., West G.M., Kovach A., Tan M.E., Suino-Powell K.M., He Y., Xu Y., Chalmers M.J. (2012). Molecular mimicry regulates ABA signaling by SnRK2 kinases and PP2C phosphatases. Science.

[53] Clough S.J., Bent A.F. (1998). Floral dip: A simplified method for *Agrobacterium*-mediated transformation of *Arabidopsis thaliana*. Plant J..

[54] Heberle H., Meirelles G.V., da Silva F.R., Telles G.P., Minghim R. (2015). InteractiVenn: A web-based tool for the analysis of sets through Venn diagrams. BMC Bioinform..

[55] Babicki S., Arndt D., Marcu A., Liang Y., Grant J.R., Maciejewski A., Wishart D.S. (2016). Heatmapper: Web-enabled heat mapping for all. Nucleic Acid Res..

