# Supplementary material for: Low Mannitol Concentrations in Arabidopsis thaliana Expressing Ectocarpus Genes Improve Salt Tolerance
Source: Plants (Basel). 2020 Nov 7;9(11):1508. doi: 10.3390/plants9111508 (PMC7695032; doi:10.3390/plants9111508)
Supplement: Supplementary file 1 [file plants-09-01508-s001.zip › plants-963387-supplementary for XML.docx]

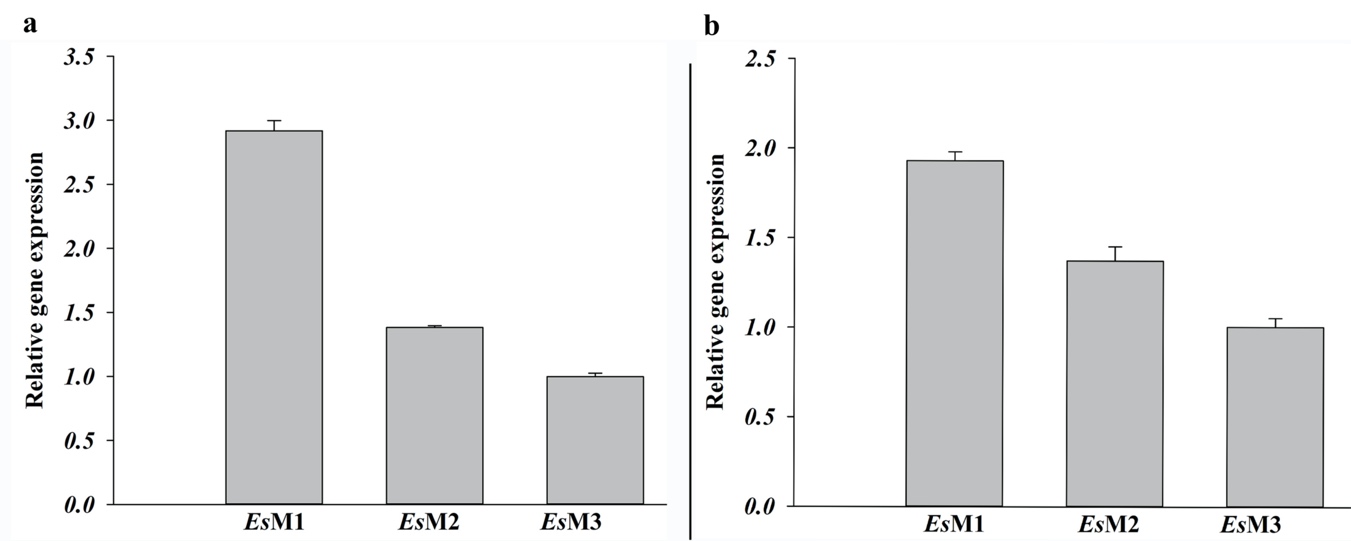


**Figure S1.** Gene expression analysis of *M1PDH1* and *M1Pase2* in transformed *A. thaliana* plants. (**a**) Relative gene expression of *M1PDH1,* (**b**) Relative gene expression of *M1Pase2.* Actin was used as endogenous control and transcript levels were normalized to individual with the lowest expression in the transgenic line *Es*M3. *Es*M1, *Es*M2 and *Es*M3, are the three transgenic lines.


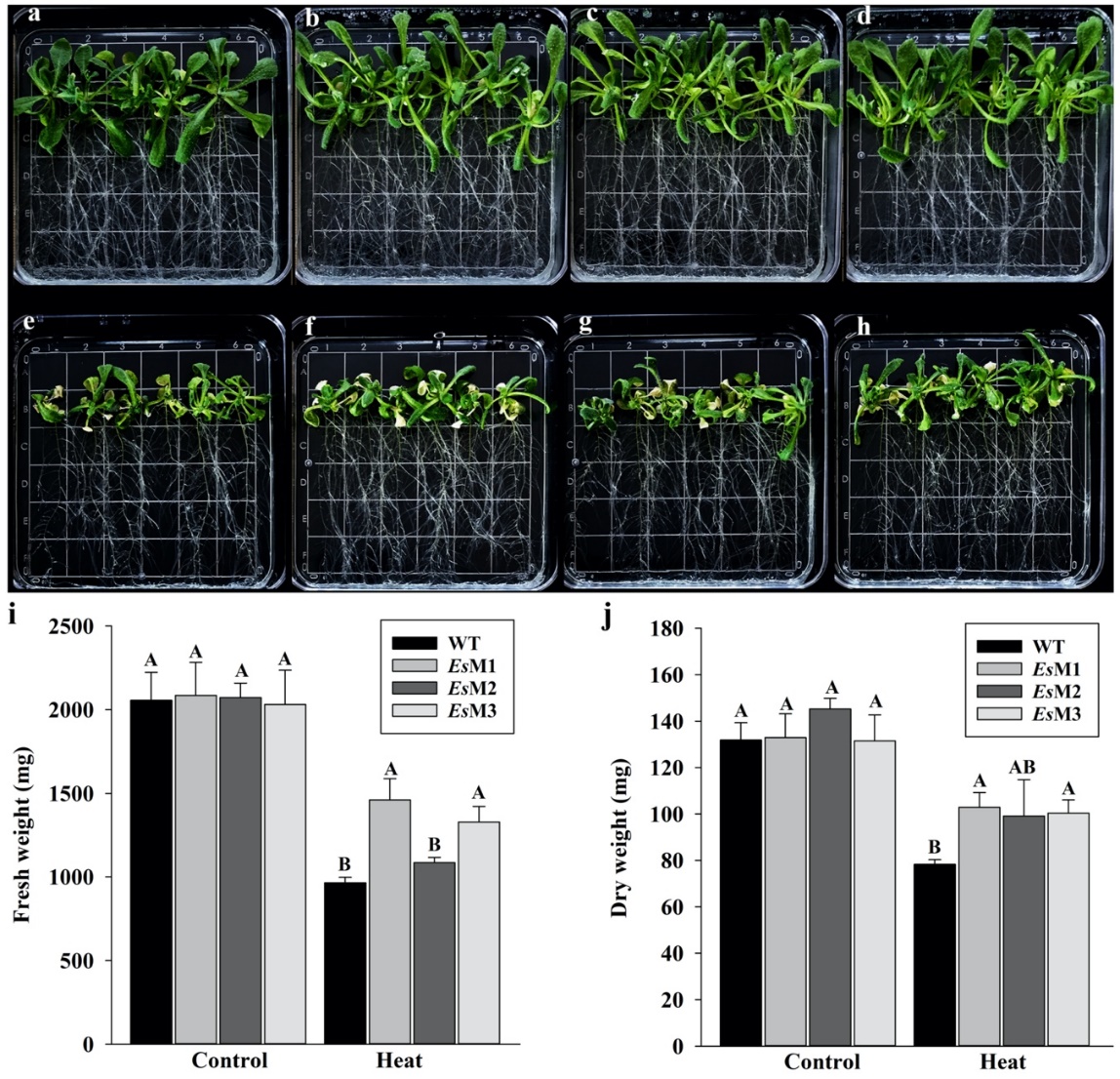


**Figure S2.** Seedlings growth, fresh weight (FW) and dry weight (DW) of the wild type and 3 independent transgenic lines (*Es*M1, *Es*M2 and *Es*M3), in the absence and presence of high temperature stress conditions. (**a**–**d**), seedlings were grown under standard conditions; (**e**–**h**) seedlings subjected to high temperature stress (40 °C for 24 h). (**a**,**e**) WT, (**b**,**f**) *Es*M1, (**c**,**g**) *Es*M2 and (**d**,**h**) *Es*M3. The 18 days old seedlings were photographed one week after being exposed to high temperature stress. Each grid has 13 mm. (**i**) fresh weight, (**j**) dry weight. Columns represents the mean and the bars the standard error (n = 9). Means and SE with the same letter are not significantly different.


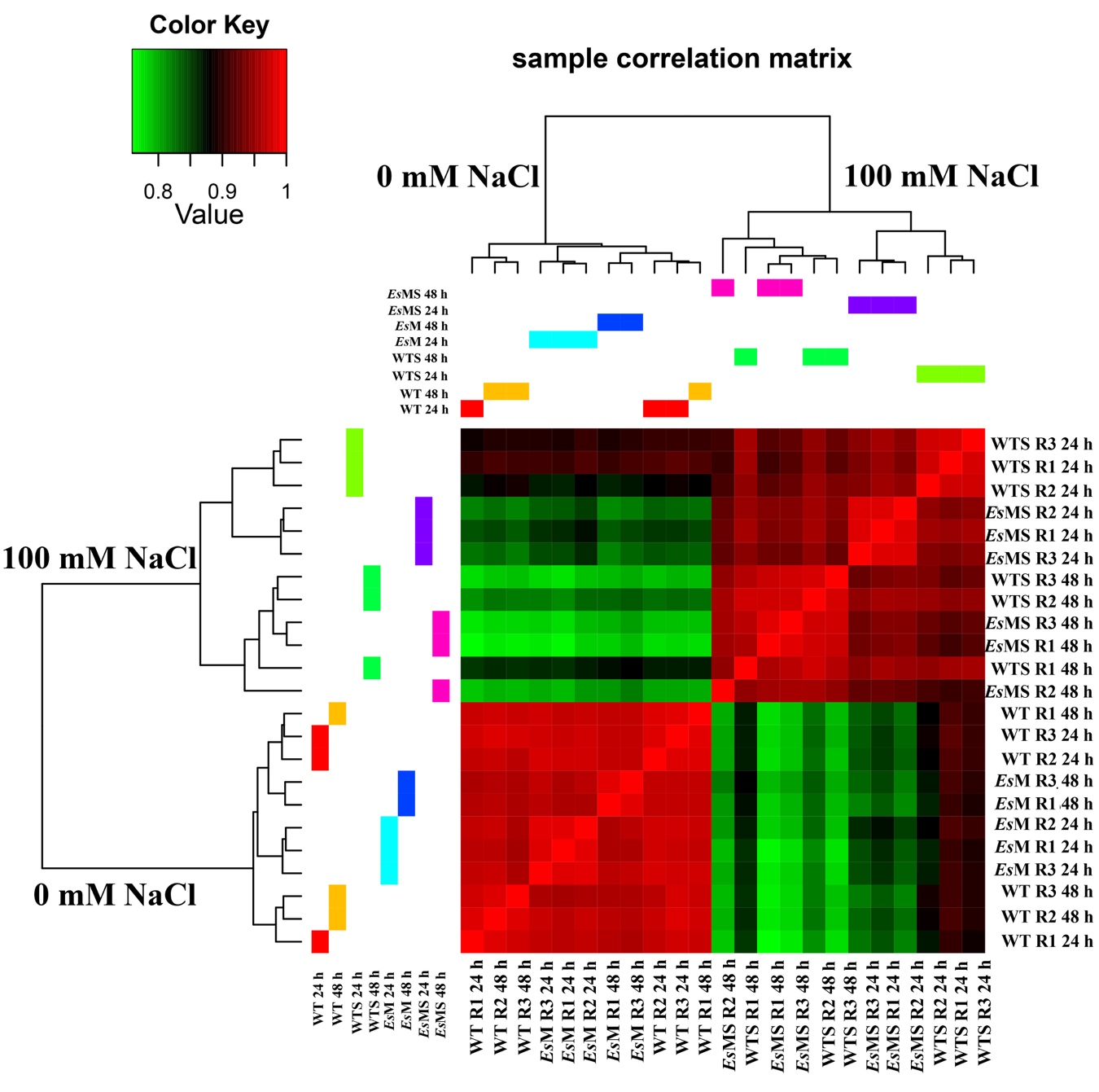


**Figure S3.** Correlation analysis showing separation of libraries in absences and presence of salinity stress at 24 h and 48 h.


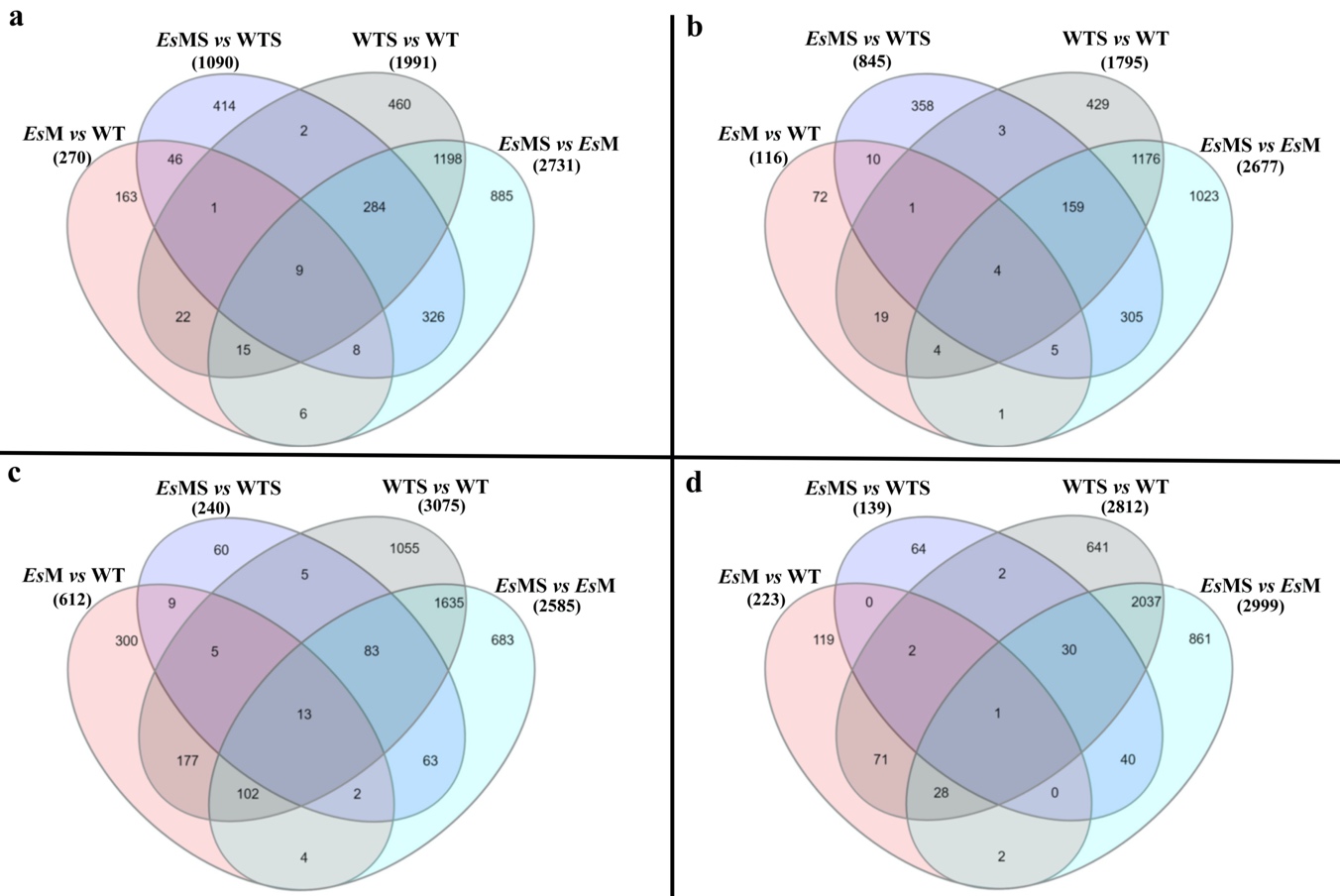


**Figure S4.** Venn diagram showing the differentially expressed genes (> 2-fold up- and down-regulated) in the mannitol transgenic line *vs* wild type, in standard (*Es*M and WT, respectively) and salinity stress conditions (*Es*MS and WTS, respectively), at 24 h and 48 h (**a**) genes that are up-regulated at 24 h (**b**) genes that are down-regulated at 24 h (**c**) genes that are up-regulated at 48 h (**d**) genes that are down-regulated at 48 h.

**Table S1.** Primers used to amplify *A. thaliana* *actin 2* and *Ectocarpus sp*. *M1PDH1* and *M1Pase2* genes*.*

| **Gene** | **Sequence 5’ to 3’** |
| --- | --- |
| *Actin*2 F | GCACCCTGTTCTTCTTACCG |
| *Actin*2 R | AACCCTCGTAGATTGGCACA |
| *M1PDH1* F | GTTCAGGCAGGGATTCCACA |
| *M1PDH1* R | GCTCTCTGTCCAACAGGCAT |
| *M1Pase2* F | AGGATAGGAAGGCTGCTGGA |
| *M1Pase2* R | GCTCAGCGAAGGTCTTGAGT |

**Table S2.** The amount of mannitol in the three double transgenic *A. thaliana* lines, determined by LC-MS.

| **Sample** | **nmol g^−1^ fresh tissue (mean and SD)** |
| --- | --- |
| WT | 0 |
| *Es*M1 | 52.68 ± 21.54 |
| *Es*M2 | 48.78 ± 0.89 |
| *Es*M3 | 42.27 ± 3.67 |

**Table S3.** Root length, number of lateral roots per cm of primary root, and leaf chlorosis of the wild type seedlings and 3 independent transgenic lines (*Es*M1, *Es*M2 and *Es*M3), grown in the presence and absence of 100 mM NaCl. Values represents percentage to wild type plants (n = 150).

|  | **Root length (%)** | **Lateral roots (%)** | **Leaf chlorosis (%)** |
| --- | --- | --- | --- |
|  | 0 mM NaCl |  |  |
| WT | 100 | 100 |  |
| *Es*M1 | 103.15 | 109.33 |  |
| *Es*M2 | 90.16 | 115.16 |  |
| *Es*M3 | 103.54 | 106.71 |  |
|  | 100 mM NaCl |  |  |
| WT | 100 | 100 | 100 |
| *Es*M1 | 108.47 | 116.54 | 43.62 |
| *Es*M2 | 104.23 | 125.98 | 37.11 |
| *Es*M3 | 115.96 | 130.31 | 41.67 |

**Table S4.** Fresh weight and dry weight of the wild type and 3 independent transgenic lines (*Es*M1, *Es*M2 and *Es*M3), grown in the absence and presence of 100 mM NaCl. Values represents percentage to wild type plants (n = 18).

|  | **FW (%)** | **DW (%)** | **FW (%)** | **DW (%)** |
| --- | --- | --- | --- | --- |
|  | Seedlings |  |  |  |
|  | 0 mM NaCl | | 100 mM NaCl | |
| WT | 100 | 100 | 100 | 100 |
| *Es*M1 | 108.77 | 111.21 | 127.68 | 126.29 |
| *Es*M2 | 106.87 | 119.39 | 135.49 | 152.64 |
| *Es*M3 | 105.08 | 109.08 | 140.14 | 142.08 |
|  |  |  |  |  |
|  | Plants |  |  |  |
|  | 0 mM NaCl | | 100 mM NaCl | |
| WT | 100 | 100 | 100 | 100 |
| *Es*M1 | 86.97 | 89.17 | 112.16 | 110.24 |
| *Es*M2 | 112.54 | 116.81 | 113.74 | 120.11 |
| *Es*M3 | 109.15 | 115.95 | 122.16 | 123.97 |

**Table S5.** Fresh weight and dry weight of the wild type seedlings and 3 independent transgenic lines (*Es*M1, *Es*M2 and *Es*M3), grown in the absence and presence of high temperature stress conditions. Values represents percentage to wild type plants (n = 9).

|  | **FW (%)** | **DW (%)** | **FW (%)** | **DW (%)** |
| --- | --- | --- | --- | --- |
|  | Control | | Heat | |
| WT | 100 | 100 | 100 | 100 |
| *Es*M1 | 101.37 | 100.8 | 151.31 | 131.43 |
| *Es*M2 | 100.78 | 110.15 | 112.44 | 126.64 |
| *Es*M3 | 98.75 | 99.75 | 137.69 | 128.19 |
